# CRISPR-PCS: a powerful new approach to inducing multiple chromosome splitting in *Saccharomyces cerevisiae*

**DOI:** 10.1038/srep30278

**Published:** 2016-08-17

**Authors:** Yu Sasano, Koki Nagasawa, Saeed Kaboli, Minetaka Sugiyama, Satoshi Harashima

**Affiliations:** 1 Department of Biotechnology, Graduate School of Engineering, Osaka University, 2-1 Yamadaoka, Suita-shi, Osaka 565-0871, Japan; 2 Department of Applied Microbial Technology, Faculty of Biotechnology and Life Science, Sojo University, Ikeda 4-22-1, Kumamoto, 860-0082, Japan

## Abstract

PCR-mediated chromosome splitting (PCS) was developed in the yeast *Saccharomyces cerevisiae.* It is based on homologous recombination and enables division of a chromosome at any point to form two derived and functional chromosomes. However, because of low homologous recombination activity, PCS is limited to a single site at a time, which makes the splitting of multiple loci laborious and time-consuming. Here we have developed a highly efficient and versatile chromosome engineering technology named CRISPR-PCS that integrates PCS with the novel genome editing CRISPR/Cas9 system. This integration allows PCS to utilize induced double strand breaks to activate homologous recombination. CRISPR-PCS enhances the efficiency of chromosome splitting approximately 200-fold and enables generation of simultaneous multiple chromosome splits. We propose that CRISPR-PCS will be a powerful tool for breeding novel yeast strains with desirable traits for specific industrial applications and for investigating genome function.

The budding yeast *Saccharomyces cerevisiae* is widely used for production of various useful compounds such as biofuels and high-value metabolites. Recently, developments in metabolic engineering and synthetic biology have raised the demand for rapid and convenient integration of multistep metabolic pathways and multiple DNAs. In yeast, most industrially useful traits, such as stress tolerance, are controlled by multiple genes^1^. As a consequence, genome engineering technologies have become increasingly important for rapid and efficient manipulation of multiple genetic loci or chromosomal regions. Chromosome engineering is one of the technologies of interest for such genomic manipulations. This approach enables large scale genomic manipulation through the alteration of chromosomes and offers a powerful means for elucidating chromosome and genome function. Additionally, it can be used for breeding useful yeast strains through the creation of a wide array of genetic variants. However, the currently available techniques for chromosome engineering are limited by their inability to manipulate multiple chromosomes simultaneously^2^.

We recently developed a novel chromosome engineering technology in *S. cerevisiae* called PCR-mediated chromosome splitting (PCS) that can be used to split a yeast chromosome at any desired genomic locus and to generate two novel functional chromosomes by addition of a centromere and telomere seed sequences^3^ based on the principle of the chromosome fragmentation technique^4^. In order to split a genomic locus by PCS, two splitting modules containing an approximately 400 bp homologous sequence upstream and downstream of the target are introduced into yeast cells. A variety of chromosome engineering technologies based on PCS have been developed, for example, PCR-mediated chromosome deletion (PCD)^5^,^6^, PCR-mediated chromosome duplication (PCDup)^7^, and genome reorganization (GReO)^8^. PCD is used to delete target chromosomal regions that do not contain essential gene(s) or synthetic lethal combinations of genes. It is a very powerful tool for investigating genetic interactions, such as identifying previously unknown synthetic lethal gene combinations^9^. PCDup is the technology that can generate segmental aneuploidy at any desired chromosomal region. GReO is a PCS based yeast breeding technology and consists of two steps. First, a yeast strain carrying multiple mini-chromosomes smaller than 50 kb is constructed by successive round of splitting. Second, based on the observation that mini-chromosomes are easily lost during culture, combinatorial loss of mini-chromosomes is induced under specific culture conditions such as high temperature or high ethanol stress to create a cell population with a variety of chromosomal constitutions and accompanying genomic diversities. Subsequently, the most adapted yeast cell subpopulation will come to dominate the culture. As stated above, PCS technology offers useful chromosome engineering tool for application not only to elucidating genome function, but also to strain breeding. Although PCS is a key technology in chromosome engineering, its low efficiency of splitting is a major drawback. In PCS, splitting is effectively limited to a single site at each transformation step. Simultaneous splitting at two or more different genomic loci has never been reported. As one round of splitting takes at least 11 days and sometimes results in failure, constructing strains with multiple chromosome splits is very time-consuming and laborious. Thus, a new chromosome splitting technology that enables simultaneous multiple chromosomes splitting would lead to rapid, convenient and reliable chromosome manipulation, which undoubtedly facilitate studies of genome function and strain breeding.

As the chromosome splitting step in PCS technology is based on the mechanism of homologous recombination, we postulated that enhancement of homologous recombination activity would be the key to increasing chromosome splitting efficiency. Double strand breaks (DSBs) are known to promote homologous recombination activity in *S. cerevisiae* by activating the DSB repair pathway^10^. In previous studies, the mating-type switching enzyme HO endonuclease^11^ or the mitochondrial I-SceI endonuclease^12^ were utilized to induce DSBs. However, these systems require the prior integration of an endonuclease recognition site before induction of the DSB, which hampers rapid and convenient DSB induction. Recently, a novel method for site-specific induction of DSBs, called the CRISPR/Cas9 system, was developed^13^,^14^,^15^. The CRISPR/Cas9 system is especially useful as a genome editing tool and is based on sequence specific DSB induction by the nuclease Cas9 in co-operation with sequence targeting guide RNA (gRNA). The determinant of target specificity is a 20 bp target sequence in the gRNA and the presence of a DNA sequence termed the protospacer adjacent motif (PAM) in the target genome next to the 20 bp target sequence. The CRISPR/Cas9 system has been shown to function in *S. cerevisiae*^16^. This system allows site-specific DSB induction without the need for prior integration of an endonuclease recognition site and is therefore a comparatively simple and rapid method for induction of DSBs. On the basis of the mechanism of CRISPR/Cas9, we speculated that induction of DSBs by this system prior to chromosome splitting would strongly increase the efficiency of the latter.

Here, we demonstrate that the combined methodology, which we call CRISPR-PCS, provides a novel genome engineering technology that is highly efficient for chromosome splitting and allows simultaneous splitting of multiple chromosomes.

## Results

### Increased splitting efficiency by CRISPR-PCS

**Figure 1:**
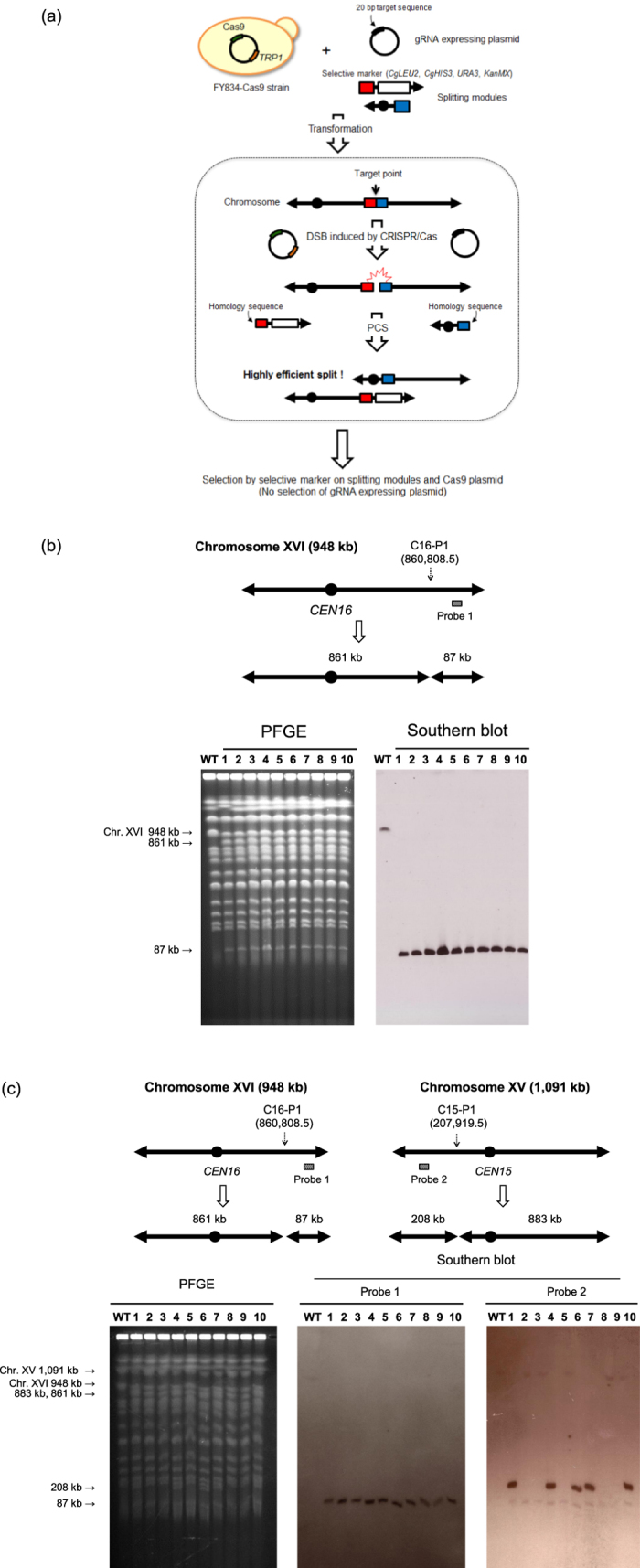
Simultaneous double splitting by CRISPR-PCS. (**a**) Outline of CRISPR-PCS. One gRNA expressing plasmid for the specific targeting site and two splitting modules per target site are introduced into the FY834-Cas9 strain, which harbors a Cas9 expressing plasmid marked by *TRP1*. The FY834-Cas9 strain was transformed with gRNA expressing plasmid with 20 bp specific target sequence and splitting modules. For splitting one genomic locus, one of the splitting modules contains either one of selective markers (*CgLEU2*, *CgHIS3*, *URA3*, and *KanMX*) and the other module contains *CEN4* as a centromere. Double strand breaks (DSBs) are induced in transformed cells by CRISPR/Cas9 near the targeted site followed by chromosome splitting by PCS. This combination of CRISPR/Cas9 and PCS is called CRISPR-PCS. After transformation, we selected by selective marker on splitting modules and Cas9 expressing plasmid. No selection of gRNA expressing plasmid was performed. Closed black circles represent the centromere. White boxes represent selective marker genes. Red and blue boxes represent the homology sequences for recombination. Arrows represent the telomere sequence. (**b**) Chromosome splitting by CRISPR-PCS. Position C16-P1 of Chr. XVI was chosen as the example. The number in parentheses represents the precise splitting point (the same shall apply hereinafter in all figures). The splitting module was marked with *CgLEU2* gene. After splitting, two chromosomes (861 kb and 87 kb) are expected to be generated. Left panel, PFGE analysis of wild type FY834-Cas9 (WT) and 10 randomly chosen transformants (lanes 1–10). Right panel, Southern blot analysis after PFGE using probe 1 for detection of the newly generated 87 kb chromosome. (**c**) Simultaneous double splitting in different chromosomes. Position C16-P1 of Chr. XVI and position C15-P1 of Chr. XV were simultaneously split by CRISPR-PCS. The splitting modules of C15-P1 and C16-P1 were marked with *CgHIS3* and *CgLEU2*, respectively. After splitting, four derived chromosomes are expected: 861 kb and 87 kb derivatives from Chr. XVI and 208 kb and 883 kb derivatives from Chr. XV. Left panel, PFGE analysis of wild type FY834-Cas9 (WT) and 10 randomly chosen transformants. Middle panel and right panel, Southern blot analysis using probe 1 for detecting the newly generated 87 kb chromosome and probe 2 for detecting the newly generated 208 kb chromosome.

We devised the CRISPR-PCS method, a combination of CRISPR/Cas9 and PCS systems, in order to carry out simultaneous and multiple chromosome splitting (Fig. 1a). Our first step was to confirm that the method increased splitting efficiency. To this end, a p414-TEF1-Cas9-CYC1t plasmid harboring a Cas9 expressing cassette under the strong constitutive *TEF1* promoter was introduced into the FY834 strain. The Cas9-expressing FY834 strain (FY834-Cas9) was used for chromosome splitting. FY834-Cas9 strain showed no significant growth defect compared with FY834 strain, suggesting that Cas9 expression is not toxic in this strain. We designed a gRNA targeting sequence near the intended split site. The genomic positions chosen for splitting in this study and the gRNA targeting sequences are shown in Supplementary Table 1. Initially, we attempted to produce a split at C16-P1 on Chr. XVI. The FY834-Cas9 strain was transformed by the simultaneous introduction of splitting modules marked with the *Candida glabrata LEU2* gene (*CgLEU2*) gene for position C16-P1 and a gRNA expression plasmid whose target sequence was located near C16-P1. After transformation, a total of 680 Leu^+^ transformants were obtained when the CRISPR-PCS system was employed (Table 1). By contrast, conventional PCS, i.e., the same transformation conditions except that no gRNA expression plasmid was added, yielded only 3 Leu^+^ transformants. We chose 10 transformants at random from those obtained by CRISPR-PCS and used Pulsed field gel electrophoresis (PFGE) and subsequent Southern blot analysis to determine whether the splitting event had occurred at the expected locus. The analysis showed that Chr. XVI (948 kb) was split into 861 kb and 87 kb products in all transformants (Fig. 1b). Thus, all the transformants were split at the expected site when CRISPR-PCS was used as the splitting method. The transformants from conventional PCS all showed a split at the expected site although the transformation frequency was significantly lower compared with CRISPR-PCS (Table 1). A similar result was obtained when C15-P1 at Chr. XV was targeted, whose splitting event was marked with *CgHIS3* gene (Table 1). We conclude that CRISPR-PCS enhanced chromosome splitting efficiency by approximately 200-fold (680 His^+^ transformants in the CRISPR-PCS and 3 His^+^ transformants in the conventional PCS). There was no indication that genomic rearrangements occurred when using CRISPR-PCS since the PFGE band pattern was unchanged except for products of the splitting event (Fig. 1b).

### Optimization of amount of gRNA expression plasmid for CRISPR-PCS

Next, we sought to identify the optimum amount of the gRNA expression plasmid for efficient transformation and splitting. We performed a similar experiment as above to split Chr. XVI at position C16-P1; the effects of 0, 0.5, 1, 3, 7.5, and 15 μg of the gRNA expression plasmid were compared by counting the number of transformants. The highest yield of transformants was obtained using 7.5 μg of the gRNA expression plasmid (Supplementary Table 2). Higher concentrations of the plasmid resulted in a decrease in transformation efficiency. As a consequence of this experiment, we used 7.5 μg of gRNA expression plasmid in all further CRISPR-PCS experiment in this study.

### Simultaneous double splitting by CRISPR-PCS

In conventional PCS, the low efficiency of splitting has meant that simultaneous splitting at different genomic loci has been unsuccessful. Using our new CRISPR-PCS approach, we attempted to induce a simultaneous split at two genomic loci on different chromosomes, namely, Chr. XV (at C15-P1) and Chr. XVI (at C16-P1). We obtained 119 His^+^ Leu^+^ transformants using CRISPR-PCS, whereas no transformants were obtained using conventional PCS (Table 1). Ten transformants obtained by CRISPR-PCS were randomly selected and analyzed for double splitting using PFGE and Southern blot analysis (Fig. 1c). Five of the transformants from CRISPR-PCS showed double splitting. All the rest of five transformants that did not have double splitting from CRISPR-PCS had only single splitting at chromosome XVI (C16-P1) confirmed by PFGE and Southern blotting. Thus, simultaneous double splitting can be induced successfully using CRISPR-PCS.

**Figure 2:**
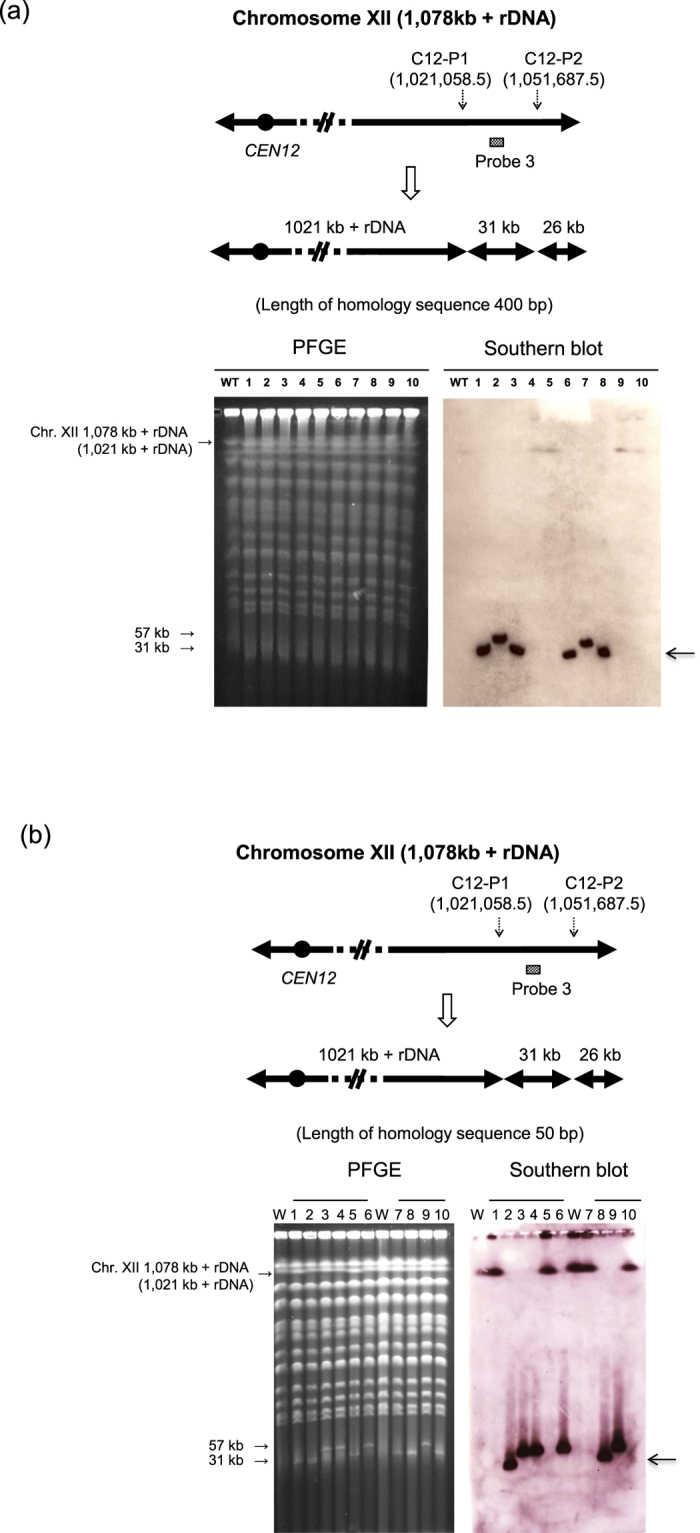
One step mini-chromosome construction by CRISPR-PCS. (**a**) One step mini-chromosome construction using CRISPR-PCS. The chromosomal region between C12-P1 and C12-P2 of Chr. XII was targeted to form a mini-chromosome. A 400 bp homology sequence was used in the splitting modules. The splitting modules of C12-P1 and C12-P2 were marked with *CgHIS3* and *CgLEU2*, respectively. Left panel, PFGE analysis of wild type FY834-Cas9 and 10 randomly chosen transformants. Right panel, Southern blot analysis after PFGE using probe 3 for detection of newly generated 31 kb mini-chromosome. The arrow beside the right panel represents the 31 kb expected mini-chromosome (lane 1, 3, 6, and 8). The 57 kb band in lane 2 and 7 show these strains have only one split at C12-P1. (**b**) One step mini-chromosome construction using CRISPR-PCS under the same experimental conditions as above except for use of a 50 bp homology sequence in the splitting module. The splitting modules of C12-P1 and C12-P2 were marked with *CgHIS3* and *CgLEU2*, respectively. The arrow beside the right panel represents the 31 kb expected mini-chromosome. The expected mini-chromosome was constructed in two strains (Lane 2 and 8). Four strains are shown to have single splitting at C12-P1 (Lane 3, 4, 6, and 9). Four strains are shown to have single splitting at C12-P2 (Lane 1, 5, 7, and 10). W. Wild type.

Next, we attempted double splitting of the same chromosome to generate a mini-chromosome in a single step. We targeted two genomic sites on Chr. XII (C12-P1 and C12-P2) to generate an approximately 30 kb mini-chromosome. In a previous study, we showed that it was feasible to generate a mini-chromosome in this region by two rounds of successive splitting^9^. Here, we obtained 118 His^+^ Leu^+^ transformants by CRISPR-PCS, while no transformants were obtained by conventional PCS (Table 2). Ten transformants from CRISPR-PCS were randomly selected for analysis by PFGE and Southern blot analysis (Fig. 2a). For the CRISPR-PCS group, four of the transformants carried the expected mini-chromosome. Concerning the transformants that did not generate the expected mini-chromosome, the reason for this event will be discussed in the Discussion section. Thus, we succeeded in constructing a mini-chromosome at one step by CRISPR-PCS. This success will encourage the use of GReO technology by facilitating the construction step of yeast strains carrying many mini-chromosomes.

In conventional PCS, the splitting modules needed for efficient activity have long homology sequences (approximately 400 bp) corresponding to the genomic target site. Preparation of the splitting module requires two rounds of amplification by overlap-extension PCR, making this preparation step laborious, time-consuming, and costly. In order to make the splitting module preparation step simpler and more reliable, we examined the efficiencies of shorter homology sequences (25, 50, and 75 bp) in the splitting module prepared by a single PCR. Using Chr. XVI (C16-P1) as the target for splitting, we found that a 50 bp homology sequence was sufficient for success by CRISPR-PCS (Supplementary Table 3). However, use of the 25 bp homology sequence drastically decreased splitting efficiency. No transformants were obtained by conventional PCS using any of the tested lengths of homology sequence (data not shown).

Based upon this observation, we next tried to construct a mini-chromosome using CRISPR-PCS with a splitting module carrying a 50 bp homology sequence. The selected target region on Chr. XII (between C12-P1 and C12-P2) was that described above for mini-chromosome construction with the 400 bp homology sequence (Table 2). The splitting module with the 50 bp homology sequence produced 139 His^+^ Leu^+^ transformants by CRISPR-PCS (Table 2). By contrast, no transformants were obtained by the conventional PCS method. We selected 10 of the 139 transformants at random and carried out PFGE and Southern blot analysis (Fig. 2b). Two of the transformants carried the expected mini-chromosome (Lane 2 and 8). In order to confirm that CRISPR-PCS is a consistently reliable method for one step mini-chromosome construction, we next targeted a Chr. IV region (between C4-P1 and C4-P2). As described for Chr. XII, the expected mini-chromosome was generated only when CRISPR-PCS was used (Table 2 and Supplementary Fig. 1). Thus, a mini-chromosome can be generated in one transformation step by CRISPR-PCS using splitting modules prepared by one step PCR. This technological advance makes CRISPR-PCS much simpler and more convenient than conventional PCS.

### Simultaneous multiple splitting by CRISPR-PCS

**Figure 3:**
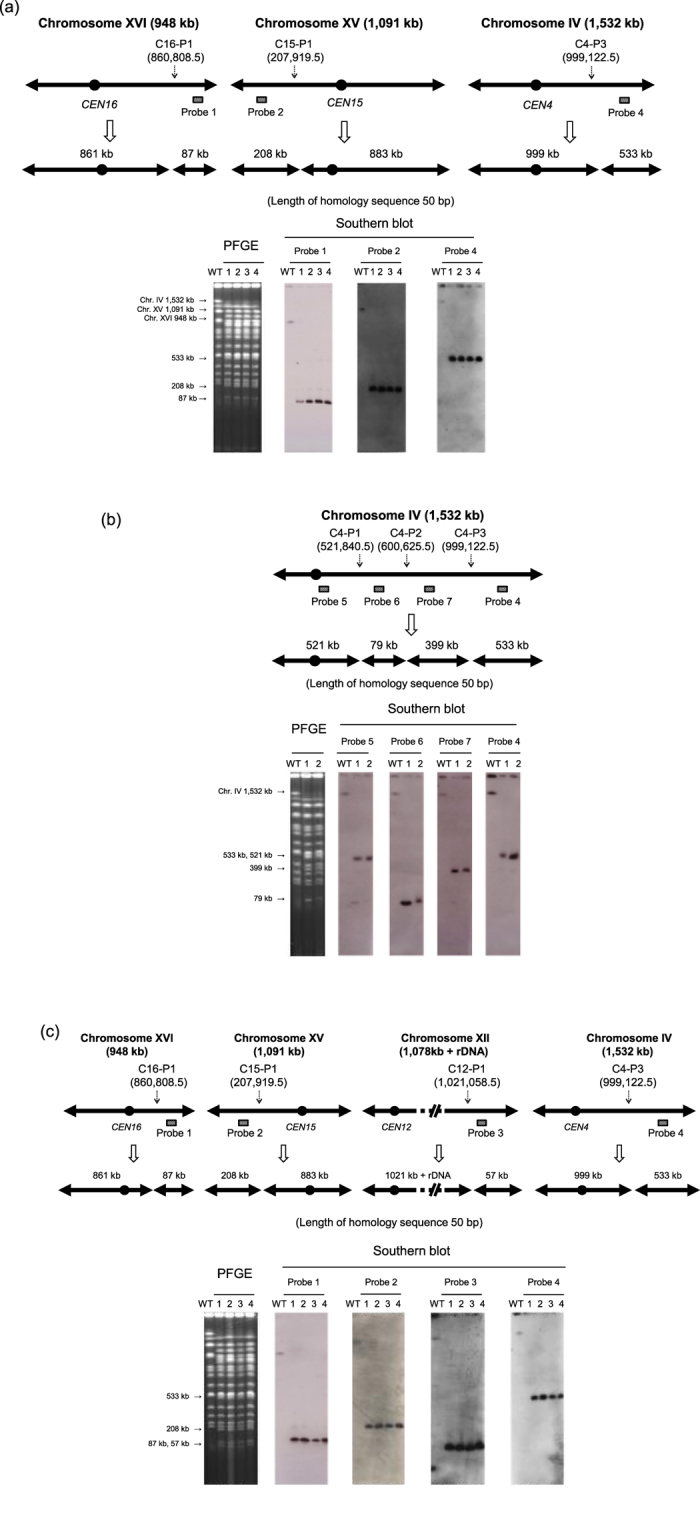
Simultaneous multiple splitting. (**a**) Simultaneous triple splitting by CRISPR-PCS. Three sites (C16-P1, C15-P1, and C4-P3) were targeted. A 50 bp homology sequence was used in the splitting module. The splitting modules of C16-P1, C15-P1, and C4-P3 were marked with *CgLEU2, CgHIS3,* and *URA3*, respectively. Wild type FY834-Cas9 and four randomly chosen transformants were subjected to PFGE and subsequent Southern blot analysis. Probes 1, 2, and 4 were used to detect Chr. XVI, Chr. XV, and Chr. IV respectively. (**b**) One step construction of four chromosomes from one chromosome. Chr. IV was targeted to split at three positions (C4-P1, C4-P2, and C4-P3). A 50 bp homology sequence was used in the splitting module. The splitting modules of C4-P1, C4-P2, and C4-P3 were marked with *CgHIS3, CgLEU2,* and *URA3*, respectively. Wild type FY834-Cas9 and two randomly chosen transformants were subjected to PFGE and subsequent Southern blot analysis. Probes 5, 6, 7, and 4 were used to detect the 522 kb, 79 kb, 398 kb, and 533 kb chromosomes, respectively. (**c**) Simultaneous quadruple splitting by CRISPR-PCS. Four sites (C16-P1, C15-P1, C12-P1, and C4-P3) were targeted. A 50 kb homology sequence was used in the splitting module. The splitting modules of C16-P1, C15-P1, C12-P1, and C4-P3 were marked with *CgLEU2, CgHIS3, KanMX*, and *URA3*, respectively. Wild type FY834-Cas9 and four randomly chosen transformants were subjected to PFGE and subsequent Southern blot analysis. Probes 1, 2, 3, and 4 were used to detect Chr. XVI, XV, XII, and IV, respectively.

We next examined whether it is feasible to induce simultaneous splits in more than one chromosome using CRISPR-PCS. Three target loci were selected: Chr. IV (at position C4-P3), Chr. XV (at position C15-P1), and Chr. XVI (at position C16-P1) (Table 3). In total, 206 Ura^+^ His^+^ Leu^+^ transformants were obtained by CRISPR-PCS with splitting modules containing 50 bp sequence homologies. No transformants were obtained by conventional PCS. PFGE and Southern blot analysis confirmed that the expected splits occurred at the three target loci in four of ten (splitting rate, 40%) randomly chosen transformants (Fig. 3a). We also performed triple splitting at C12-P1, C15-P1, and C16-P1. After transformation, 414 His^+^ Leu^+^ G418^r^ colonies were obtained. Among them, we randomly picked up 38 colonies and found that 6 colonies (splitting rate, 16%) were predicted to have triple splitting confirmed by colony direct PCR (data not shown).

Having demonstrated that CRISPR-PCS can generate a mini-chromosome efficiently by one-step transformation, we attempted to simultaneously induce three splits in one chromosome to produce four chromosomes at a time. Three genomic loci on Chr. IV were targeted, namely C4-P1, C4-P2, and C4-P3. We obtained Ura^+^ His^+^ Leu^+^ transformants and showed that some of these carried the expected four generated chromosomes (Table 3 and Fig. 3b).

We also attempted to induce splits at four genomic loci on different chromosomes, namely, C4-P3, C12-P1, C15-P1, and C16-P1. As in the above experiments, Ura^+^ His^+^ Leu^+^ G418^r^ transformants were recovered and some were shown by PFGE and Southern blot analysis to contain the expected derived chromosomes (Table 3 and Fig. 3c). We therefore concluded that multiple (quadruple) simultaneous splits were feasible using CRISPR-PCS; this outcome was never obtained using conventional PCS.

### Karyotype analysis of transformants that were not split at expected site by CRISPR-PCS

In this study, we showed that CRISPR-PCS can split multiple chromosomes simultaneously. However, not all the transformants did not have an expected splitting. In order to investigate what had happened in such unexpected transformants, we performed simultaneous splitting at position C16-P1 of Chr. XVI and position C15-P1 of Chr. XV by CRISPR-PCS with 50 bp homology sequence in the splitting modules. The splitting modules of C15-P1 and C16-P1 were marked with *CgHIS3* and *CgLEU2*, respectively. Among His^+^ Leu^+^ transformants, we isolated 20 transformants that had been split at Chr. XVI but did not have spitting at Chr. XV as confirmed by colony direct PCR (data not shown). These 20 transformants were subjected to karyotype analysis by PFGE and subsequent Southern blot analysis (Supplementary Fig. 2). Southern blot analysis using probe 2 clearly shows that all these transformants do not have splitting at Chr. XV. Surprisingly, when *CgHIS3* gene probe was used, *CgHIS3* marker gene was detected at the same chromosome in all transformants, suggesting that *CgHIS3* gene was not integrated at random but integrated into a specific point. Judging from the mobility, the chromosome in which *CgHIS3* gene was detected seemed likely to be newly generated 861 kb chromosome derived from Chr. XVI, although the precise location of the integration is unclear. We also isolated transformants that had been split at Chr. XV but did not have spitting at Chr. XVI. Similar to the result stated above, *CgLEU2* marker gene was detected at the same chromosome in all the transformants (data not shown). Taken together, in a transformant that did not have expected splitting, splitting modules were not integrated randomly in the genome but integrate into a specific genomic locus.

## Discussion

In this study, we developed a new chromosome engineering technology by integrating the previously described CRISPR/Cas9 and PCS systems to give a highly efficient approach for controlled chromosome splitting. We named this new method CRISPR-PCS. It enables simultaneous chromosome splitting at multiple loci. The high chromosome splitting efficiency of the new method is a consequence of the DSBs induced by CRISPR/Cas9 that stimulate an increased rate of homologous recombination. Using conventional PCS, we never succeeded in generating simultaneous double splits. CRISPR-PCS, however, has made this possible and even allowed the introduction of simultaneous quadruple splits.

In typical CRISPR/Cas9-mediated genome editing experiment, marker selection is conducted for Cas9 and gRNA plasmid after transformation while marker genes are not usually attached and selected for donor DNA. In contrast, in the CRISPR-PCS experiment, we selected by marker genes in splitting modules but selection for gRNA expression plasmid was not conducted. The reason for this was as follows: i) because the main purpose of this study is to develop a novel chromosome engineering technology enabling simultaneous and multiple chromosome splitting. For that purpose, we added marker genes in splitting modules to facilitate the isolation of chromosome split strain. ii) If we chose to select by gRNA plasmid, we need to use different selection makers in all gRNA plasmids when we intend to perform multiple splitting. This is not suitable for practical use especially when we try to perform multiple splitting in various combinations. iii) When we select transformants by marker gene (*URA3*) in gRNA plasmid, the number of transformants was decreased to one fourth compared with those obtained by selection by marker genes in splitting modules (data not shown), suggesting that continual targeted endonuclease activity is lethal for cell.

The splitting efficiency when targeting a single site was increased by approximately 200-fold by CRISPR-PCS (Table 1). This drastic increased splitting efficiency is probably the reason for the success of multiple splitting. In this study, we succeeded in up to quadruple splitting. A previous study reported that *S. cerevisiae* can take up 25 different DNA fragments^17^. This suggests that once a yeast cell acquires competency, it is able to take up multiple foreign DNAs. In addition, DiCarlo *et al.* reported that foreign donor DNA was integrated with a near 100% frequency at the target site when CRISPR/Cas9-mediated DSBs were introduced^16^. Based on these reports, simultaneous splitting of more multiple sites such as quintuple and sextuple splitting might be possible.

In multiple splitting, some transformants did not show expected karyotype. For example, in Fig. 1c, among 10 transformants obtained from double splitting experiment at C16-P1 and C15-P1, 5 transformants showed the expected double splitting but all the rest of 5 transformants showed splitting only at C16-P1, not at C15-P1. In this study, we investigated what happened in such “unexpected” transformants by karyotype analysis (Supplementary Fig. 2). The result strongly suggested that splitting modules are not integrated non-specifically but specifically. Although we have not yet specified where and how this specific integration had occurred, it is likely that recombination through the common sequence between splitting modules such as the common primer annealing site for construction of splitting modules might happen. Revealing these information would greatly improve CRISPR-PCS by increasing frequency of expected splitting event, leading to a marker-less splitting and more multiple splitting, *e.g.* quintuple and sextuple splitting.

In the conventional PCS, we prepared splitting modules with 400 bp homology sequence. This preparation step is laborious and time-consuming. In the CRISPR-PCS, 50 bp homology sequence in the splitting module is enough to split multiple chromosomes. This advancement makes manipulation of chromosome splitting much faster and much more convenient, although the efficiency of mini-chromosome generation is slightly decreased especially in multiple splitting. We want to emphasize that we can obtain multiple splitting strain by checking a practical number of colonies with this much simplified method. For example, when we perform triple splitting at different chromosomes, the frequency of expected triple splitting was 16~40%. This suggests that if we check 10 transformants, at least one transformants are expected to be obtained. This is practically sufficient for an application use.

In conventional PCS, one experiment takes at least 11 days (including the 6 day confirmation step by PFGE and Southern blot analysis). Furthermore, these experiments sometimes end in failure because of the low splitting efficiency and level of skill of the user. Splitting multiple sites sequentially by conventional PCS takes 19 days, 27 days, and 35 days for two, three, and four sites, respectively. By contrast, CRISPR-PCS takes only 11 days to perform quadruple splitting, including the confirmation period. This considerable reduction in time requirements for generating multiple splits greatly enhances the usability and applicability of CRISPR-PCS for chromosome engineering.

In a previous study, we developed a novel breeding strategy, GReO^8^, that provides a powerful approach for yeast strain breeding and also for elucidation of the relationship between chromosomal constitution and phenotype. However, GReO has a bottleneck stage caused by the need to generate mini-chromosomes. As described above, even one round of splitting takes a relatively long time using conventional PCS. This bottleneck can be overcome through CRISPR-PCS which allows construction of strains with many mini-chromosomes much more rapidly than PCS. Thus, CRISPR-PCS will facilitate the usability of GReO, and will enable selections of strains with the best performance under specific fermentation conditions in a relatively short time period.

PCS technology has been applied in many chromosomal manipulation experiments such as to induce chromosomal deletions^5^ or chromosomal duplications^7^. Such studies will in future be able to use CRISPR-PCS to produce simultaneous deletions and duplications of multiple chromosomal regions.

Recently, several multiplexed genome engineering technologies based on the CRISPR/Cas9 system have been reported in yeast^18^,^19^,^20^. These novel technologies enable simultaneous multiplexed gene disruption and genetic modification. However, these methods only improve manipulation at the gene scale not the chromosomal scale. Our new method based on CRISPR/Cas9 allows multiplex engineering technology on a chromosomal scale. Various chromosome engineering techniques based on the CRISPR/Cas9 system have been developed and used in human and mouse cells to induce chromosome translocations, inversions, and deletions^21^,^22^. To date, however, site-directed simultaneous manipulation of multiple intact chromosomes has not been feasible in any organism. The CRISPR-PCS method developed and described here is a pioneer technology that offers a means for multiple chromosome manipulations not only in yeast, but also in higher organism such as mammals.

In conclusion, CRISPR-PCS enables simultaneous and multiple chromosome splitting in the budding yeast *S. cerevisiae*. It will be a powerful tool not only for breeding of yeasts exhibiting desired traits for specific industrial applications, but also for investigation of genome function.

## Methods

### Strains and media

The FY834 strain (*MAT*α *ura3-52 his3*Δ*200 leu2*Δ*1 lys2*Δ*202 trp1*Δ*63*) was used in this study^23^. FY834 cells harboring a p414-TEF1-Cas9-CYC1t plasmid were named FY834-Cas9, and used as the host strain for the CRISPR-PCS experiment. *Escherichia coli* DH5α was used for plasmid construction and propagation; *E. coli* recombinant strains were grown in Luria-Bertani (LB) medium containing 50 μg/ml ampicillin. Yeast cells were grown in YPDA medium consisting of 5% YPD broth (Sigma Aldrich) and 0.04% adenine (Wako) and in SC medium consisting of 0.67% yeast nitrogen base without amino acids (Difco), 0.2% drop out mix, and 2% glucose. SC medium lacking specific amino acids was used for auxotrophic marker selection. For solid media, 2% agar was used to solidify the medium; when required, 200 mg/L G418 was added.

### CRISPR/Cas9 system in yeast

The *Streptococcus pyogenes* Cas9 expressing plasmid (p414-TEF1p-Cas9-CYC1t) and gRNA expressing plasmid (p426-SNR52p-gRNA.CAN1.Y-SUP4t) were purchased from the AddGene repository (http://www.addgene.org). The p414-TEF1p-Cas9-CYC1t and the p426-SNR52p-gRNA.CAN1.Y-SUP4t were gifts from George Church (Addgene plasmid #43802 and #43803, respectively)^16^.

### Construction of gRNA expression plasmids

A gRNA expression plasmid targeting a specific genomic locus was constructed by the SLIC technique^24^ with some modifications. First, PCR was performed using a primer pair and p426-SNR52p-gRNA.CAN1.Y-SUP4t as the template to yield a 20 bp overlapping sequence. The oligonucleotide primers used for construction of the gRNA expression plasmid are listed in Supplementary Table 4. The amplified product was treated with DpnI to remove the template plasmid, and then gel purified. The terminal sequence of the fragment was resected with T4 DNA polymerase (NEB), annealed, and introduced into *E. coli*. The plasmid was propagated in *E. coli* cells and extracted. Finally the extracted plasmid was checked by sequencing analysis and used for the experiment.

As the p426-SNR52p-gRNA.CAN1.Y-SUP4t plasmid contains a *URA3* marker gene, we could not use *URA3* as the selectable marker in the splitting module. In order to construct a gRNA expression plasmid lacking the *URA3* auxotrophic marker, we performed PCR using the primer set URA3-deletion-forward and URA3-deletion-reverse, with p426-SNR52p-gRNA.CAN1.Y-SUP4t as the template. Using the same SLIC method as above, the pgRNA-URA3-deletion plasmid, lacking the *URA3* selection marker cassette from p426-SNR52p-gRNA.CAN1.Y-SUP4t, was constructed. The pgRNA-URA3-deletion plasmid was used as the template to construct a gRNA expression plasmid lacking the *URA3* marker.

### CRISPR-PCS

The details of the conventional PCS technology for chromosome splitting has been described previously^3^,^25^. The method of yeast transformation was lithium acetate method^26^. Briefly, two splitting modules are introduced into a yeast cell to obtain a “splitting strain”. Each module contains either a centromere (*CEN4*) or selective marker (*CgHIS3*, *CgLEU2*, *URA3*, or *KanMX*) in addition to a telomere seed sequence (six copies of a 5′-CCCCAA-3′). The p3121 plasmid^25^ was used as the template to add a centromere to the module. The centromeric *CEN4* sequence was added to one of the two modules so that the resulting new chromosomes possessed one centromere. The other module contained one of the selective marker genes. Plasmids p3009, p3008, p3276, and p1463^9^,^27^, were used as the template plasmid for PCR using the CA primer and loxP cassette primer to prepare the splitting module containing *CgHIS3*, *CgLEU2*, *URA3*, and *KanMX*, respectively. In CRISPR-PCS, a gRNA expressing plasmid was added in addition to two splitting modules. The amount of each gRNA expression plasmid added for transformation was 7.5 μg. After transformation, SC medium lacking appropriate amino acids was used for selection of transformants harboring the marker gene obtained from the splitting module. The gRNA expression plasmid was not selected on the selection plate after transformation meaning that gRNA expression was transient. The outline of CRISPR-PCS is illustrated in Fig. 1a.

Splitting modules with a small homology sequence (25, 50, or 75 bp) were constructed by one step PCR. The CA primer and a counterpart primer (*e.g.* C4-P1-left-50 bp) were used and the appropriate plasmid (p3009, p3008, p3276, or p1463) was used as the template depending on the auxotrophic marker gene that we intended to add.

### Pulsed field gel electrophoresis (PFGE) and Southern hybridization

Chromosomal DNAs from *S. cerevisiae* cultured in YPDA medium were embedded in agarose plugs as described by Sheehan and Weiss^28^. Chromosomes were separated by CHEF-DR^®^ III pulsed field gel electrophoresis system (Bio-Rad) on 1% gel in 0.5x TBE (Tris-borate-EDTA) buffer at 14 °C. After staining with ethidium bromide, DNA was transferred onto Hybond^TM^-N^+^ membrane (GE Healthcare) by capillary blotting. Probe labelling, hybridization, and signal detection were carried out by ECL Direct^TM^ nucleic acid labeling and detection system (GE Healthcare). The oligonucleotide primers used for amplifying DNA fragments for probes in Southern hybridization are shown in Supplementary Table 4.

## Additional Information

**How to cite this article**: Sasano, Y. *et al.* CRISPR-PCS: a powerful new approach to inducing multiple chromosome splitting in *Saccharomyces cerevisiae. Sci. Rep.* **6**, 30278; doi: 10.1038/srep30278 (2016).

## Supplementary Material

Supplementary Information

## Figures and Tables

**Table 1:** CRISPR-PCS enhances chromosome splitting efficiency.

Splitting point^a^	CRISPR -PCS^b^	No. of transformants	No. of transformants subjected to karyotype analysis	No. of transformants with expected splitting
C15-P1 (*CgHIS3*)	+	214	10	10
	−	1	1	1
C16-P1 (*CgLEU2*)	+	680	10	10
	−	3	3	3
C15-P1 (*CgHIS3*)C16-P1 (*CgLEU2*)	+	119	10	5
	−	0	0	0

^a^One of the two splitting modules for each splitting site contains a marker gene described in parentheses.

^b^Chromosome splitting was carried out with (+) or without (−) a gRNA expression plasmid in addition to splitting modules.

**Table 2:** One step construction of mini-chromosome by CRISPR-PCS.

Mini-chromosome region^a^^,^^b^	CRISPR -PCS	Length of homology sequence	No. of transformants	No. of transformants subjected to karyotype analysis	No. of transformants with expected splitting
C12-P1 (*CgHIS3*) ~ C12-P2 (*CgLEU2*)	+	400	118	10	4
	−	400	0	0	0
C12-P1 (*CgHIS3*) ~C12-P2 (*CgLEU2*)	+	50	139	10	2
	−	50	0	0	0
C4-P1 (*CgHIS3*) ~C4-P2 (*CgLEU2*)	+	50	188	30	3
	−	50	0	0	0

^a^Mini-chromosome was constructed between two splitting points.

^b^One of the two splitting modules for each splitting site contains a marker gene described in parentheses.

**Table 3:** Simultaneous multiple chromosome splitting by CRISPR-PCS.

Splitting point^a^	CRISPR -PCS	Length of homology sequence	No. of transformants	No. of transformants subjected to karyotype analysis	No. of transformants with expected splitting
C4-P3 (*URA3*) C15-P1 (*CgHIS3*) C16-P1(*CgLEU2*)	+	50	206	10	4
	−	50	0	0	0
C12-P1 (*KanMX*) C15-P1 (*CgHIS3*) C16-P1(*CgLEU2*)	+	50	414	38^b^	6^b^
	−	50	0	0	0
C4-P1 (*CgHIS3*) C4-P2(*CgLEU2*) C4-P3 (*URA3*)	+	50	88	10	2
	−	50	0	0	0
C4-P3 (*URA3*) C12-P1(*KanMX*) C15-P1 (*CgHIS3*) C16-P1(*CgLEU2*)	+	50	70	20	4
	−	50	0	0	0

^a^One of the two splitting modules for each splitting site contains a marker gene described in parentheses.

^b^Confirmation of expected splitting event was performed by colony direct PCR.
