# Supplementary material for: CRISPR-PCS: a powerful new approach to inducing multiple chromosome splitting in Saccharomyces cerevisiae
Source: Sci Rep. 2016 Aug 17;6:30278. doi: 10.1038/srep30278 (PMC4987674; doi:10.1038/srep30278)
Supplement: Supplementary Information [file srep30278-s1.pdf]

CRISPR-PCS: a powerful new approach to inducing multiple chromosome splitting in *Saccharomyces cerevisiae*

Yu Sasano, Koki Nagasawa, Saeed Kaboli, Minetaka Sugiyama and Satoshi Harashima

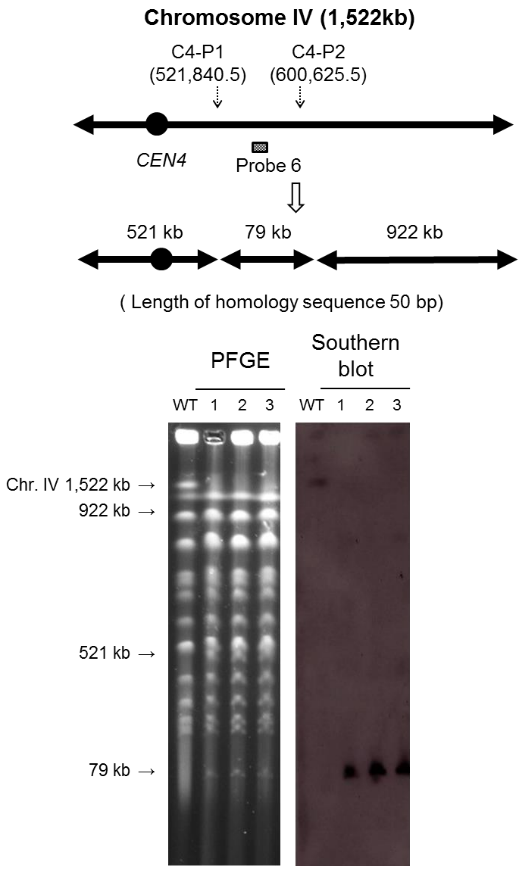

Supplementary Fig. 1. One step chromosome construction by CRISPR-PCS. The chromosomal region between C4-P1 and C4-P2 of Chr. IV was targeted to produce a mini-chromosome. A 50 bp homology sequence was used in the splitting modules. The splitting modules of C4-P1 and C4-P2 were marked with *CgHIS3* and *CgLEU2*, respectively. Left panel, PFGE analysis of wild type FY834-Cas9 and three randomly chosen transformants. Right panel, Southern blot analysis after PFGE using probe 6 for detection of the newly generated chromosome.

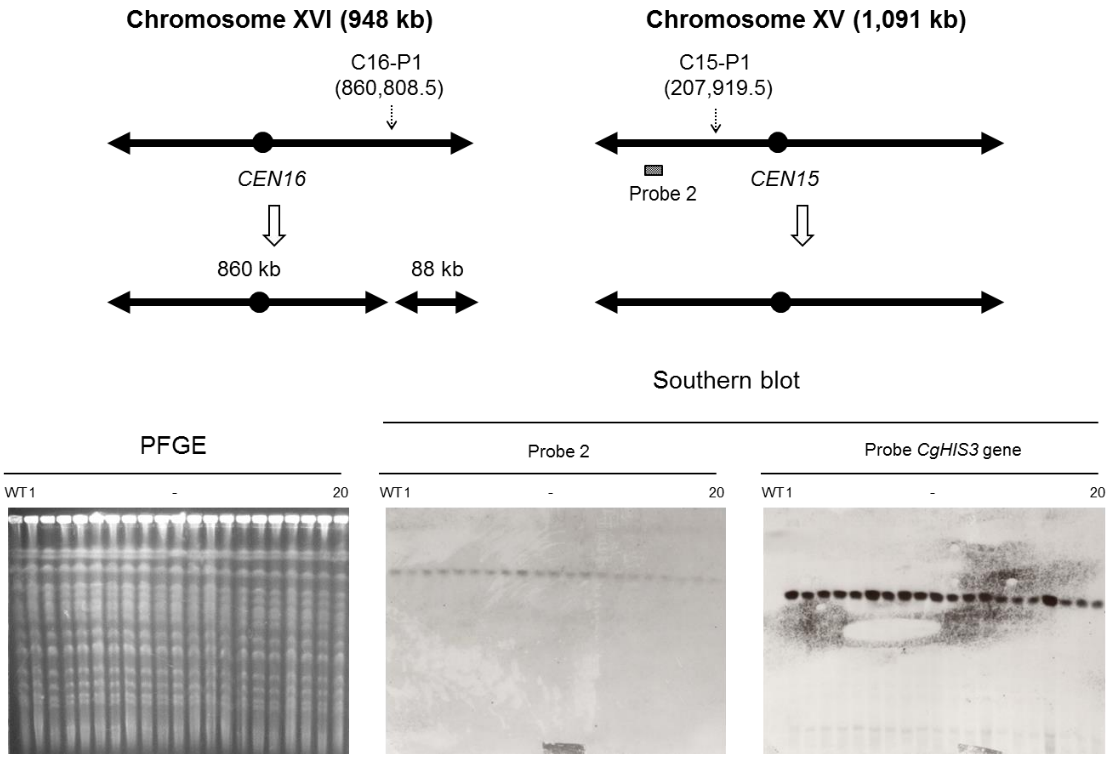

Supplementary Fig. 2. Karyotype analysis of transformants that were not split at Chr. XV. Simultaneous double splitting in different chromosomes was performed at the position C16-P1 of Chr. XVI and position C15-P1 of Chr. XV by CRISPR-PCS. The splitting modules of C15-P1 and C16-P1 were marked with *CgHIS3* and *CgLEU2*, respectively. A 50 bp homology sequence was used in the splitting modules. Among Leu^+ His^+ Transformants, we isolated 20 transformants that did not have expected splitting at Chr. XV checked by colony direct PCR. Left panel, PFGE analysis of wild type FY834-Cas9 and 20 transformants that were not split at Chr. XV; Middle panel, Southern blot analysis after PFGE using probe 2; Right panel, Southern blot analysis after PFGE using *CgHIS3* gene probe. *CgHIS3* gene probe was prepared by PCR using oligonucleotide pairs loxP cassette and short CA primer and p3009 as a template.

Table S1. Positions of splitting points on different chromosomes

**Name**	**Chromosome**	**Nucleotide position**	**gRNA targeting sequence (5`-3`)** [a]
C4-P1	Chr. IV	521,840.5	AAAACGATCACGCCGGTGACAGG
C4-P2	Chr. IV	600,625.5	GCTGGGGTAGAACTAGAGTAAGG
C4-P3	Chr. IV	999,122.5	ACGTGGTAGAACGTTAGGTGAGG
C12-P1	Chr. XII	1,021,058.5	AATAAAAGACGTGGCGCTCACGG
C12-P2	Chr. XII	1,051,687.5	TGTGACCCTCAAAGTTTGGTTGG
C15-P1	Chr. XV	207,919.5	TTTCATCAACACCAGCCTATGGG
C16-P1	Chr. XVI	860,808.5	GGGAAGAATACAACGCTCAACGG

[a] PAM sequence is underlined.

Table S2. Relationship between the amount of gRNA expression plasmid and splitting efficiency

Splitting point	Amount of gRNA expression plasmid (μg)	No. of transformants	No. of transformants subjected to karyotype analysis	No. of transformants with expected splitting
C16-P1	0	36	5	4
	0.5	45	5	5
	1	84	5	5
	3	165	5	5
	7.5	235	5	5
	15	108	5	5

Table S3. Relationship between length of homology sequence in splitting module and splitting efficiency in CRISPR-PCS

**Splitting point**	**Length of homology sequence (bp)**	**No. of transformants**	**No. of transformants subjected to karyotype analysis**	**No. of transformants with expected splitting**
C16-P1	75	451	5	5
	50	610	5	5
	25	28	5	5

Table S4. Oligonucleotide primers used in this study

Name	Sequence (5`-3`)
**For gRNA expression plasmid construction**	
C4-P1-forward	AAAACGATCACGCCGGTGACGTTTTAGAGCTAGAAATAGCAAG
C4-P1-reverse	GTCACCGGCGTGATCGTTTTGATCATTTATCTTTCACTGCGGA
C4-P2-forward	GCTGGGGTAGAACTAGAGTAGTTTTAGAGCTAGAAATAGCAAG
C4-P2-reverse	TACTCTAGTTCTACCCCAGCGATCATTTATCTTTCACTGCGGA
C4-P3-forward	ACGTGGTAGAACGTTAGGTGGTTTTAGAGCTAGAAATAGCAAG
C4-P3-reverse	CACCTAACGTTCTACCACGTGATCATTTATCTTTCACTGCGGA
C12-P1-forward	AATAAAAGACGTGGCGCTCAGTTTTAGAGCTAGAAATAGCAAG
C12-P1-reverse	TGAGCGCCACGTCTTTTATTGATCATTTATCTTTCACTGCGGA
C12-P2-forward	TGTGACCCTCAAAGTTTGGTGTTTTAGAGCTAGAAATAGCAAG
C12-P2-reverse	ACCAAACTTTGAGGGTCACAGATCATTTATCTTTCACTGCGGA
C15-P1-forward	TTTCATCAACACCAGCCTATGTTTTAGAGCTAGAAATAGCAAG
C15-P1-reverse	ATAGGCTGGTGTTGATGAAAGATCATTTATCTTTCACTGCGGA
C16-P1-forward	GGGAAGAATACAACGCTCAAGTTTTAGAGCTAGAAATAGCAAG
C16-P1-reverse	TTGAGCGTTGTATTCTTCCCGATCATTTATCTTTCACTGCGGA
URA3-deletion-forward	CACGTTCTTTAATAGTGGACTCGCACTCTCAGTACAATCTGC
URA3-deletion-reverse	GAGTCCACTATTAAAGAACGTG
**For construction of splitting module**	
CA primer	CCCCAACCCCAACCCCAACCCCAACCCCAACCCCAAAGGCCACTAGTGGATCTGAT
loxP cassette	GGCCGCCAGCTGAAGCTTCG
C4-P1-left-50bp	CGTTACAAGAACACTTTATAGCATTATGTTCATTAAAAACGATCACGCCGGGCCGCCAGCT GAAGCTTCG

C4-P1-right-50bp	TAATGGATCATAACCTTTGATTCTCCAGTCTTCCAAGCGTTTCCTGTCACGGCCGCCAGCT GAAGCTTCG
C4-P2-left-50bp	CCCAGTCGCATTATTTTTAGATTGGCCGTAGGGGCTGGGGTAGAACTAGAGGCCGCCAGC TGAAGCTTCG
C4-P2-right-50bp	GTCAGGTATTTATATGACAGTTCAAAAGAGGGCAGAGCAATGTTCCTTACGGCCGCCAGC TGAAGCTTCG
C4-P3-left-50bp	CAAGCTCCTGTATTTGCTCTTTACTACAGGAAGACGTGGTAGAACGTTAGGGCCGCCAGC TGAAGCTTCG
C4-P3-right-50bp	AAGTCTGGTACCCTTCCTATTAGTGTATGTCAAGTCAACATATTCCTCACGGCCGCCAGCT GAAGCTTCG
C12-P1-left-forward	CTCTTCCAGATGAAGAGCATG
C12-P1-left-inverse	CTGCAGCGTACGAAGCTTCAGCTGGCGGCCCGCTCACGGAAGCGAATTGA
C12-P1-right-forward	CTGCAGCGTACGAAGCTTCAGCTGGCGGCCCCACGTCTTTTATTTACTGGT
C12-P1-right-inverse	TGGAAATATACCGTCTCAGC
C12-P2-left-forward	CTTGGGATGTTGATAAGCCT
C12-P2-left-inverse	CTGCAGCGTACGAAGCTTCAGCTGGCGGCCTGGACAATCTTAAAGTCGGG
C12-P2-right-forward	CTGCAGCGTACGAAGCTTCAGCTGGCGGCCACCAAACTTTGAGGGTCACA
C12-P2-right-inverse	GGGCATTACTAATGGAAGAC
C12-P1-left-50bp	CTTGAATGTGGATTTTTCTAATTTTACTGCTCAATTCGCTTCCGTGAGCGGGCCGCCAGCT GAAGCTTCG
C12-P1-right-50bp	AGTTCAGAAAGTTCTGATGAAGCAATAATACCAGTAAATAAAAGACGTGGGGCCGCCAGC TGAAGCTTCG
C12-P2-left-50bp	GGCTAACGTTACTAACTTTGTTTTCAATTTCCCGACTTTAAGATTGTCCAGGCCGCCAGCT GAAGCTTCG
C12-P2-right-50bp	TAACACTTTTTTAAGGGCAAGTAATAGATCTGTGACCCTCAAAGTTTGGTGGCCGCCAGCT

	GAAGCTTCG
C15-P1-left-forward	GTCCGCAAATATCACCCATG
C15-P1-left-inverse	CTGCAGCGTACGAAGCTTCAGCTGGCGGCCAGGCTGGTGTTGATGAAAGC
C15-P1-right-forward	CTGCAGCGTACGAAGCTTCAGCTGGCGGCCATGGGCTGTGGTTAGGAAGA
C15-P1-right-inverse	TATGGTACAACACCGATGCC
C15-P1-left-50 bp	TGAGTTGCTGATATAATTATGAGTGGATTTGCTTTCATCAACACCAGCCTGGCCGCCAGCT
	GAAGCTTCG
C15-P1-right-50 bp	ATCAACTATCCGAAATGCACGACGAGTTCTTCTTCCTAACCACAGCCCATGGCCGCCAGCT
	GAAGCTTCG
C16-P1-left-forward	TCCAACGTTGCCATCGTTGG
C16-P1-left-reverse	CTGCAGCGTACGAAGCTTCAGCTGGCGGCCCGTTGTATTCTTCCCCCCAC
C16-P1-right-forward	CTGCAGCGTACGAAGCTTCAGCTGGCGGCCCTCAACGGAAGAGGAAGCTC
C16-P1-right-reverse	ACCTTCTCAATCTGTGCTTACT
C16-P1-left-75 bp	GCTGCCCCACAGCGGGAAGGCCCTTCAAGGGGTAGCGGCATCGCCTAGTAGCACTGTGG
	GGGGAAGAATACAACGGGCCGCCAGCTGAAGCTTCG
C16-P1-right-75 bp	TTGTTAATTCAAAATGTTATCCTTGAAACATAGAAGGTGCTGGCTGCATCTCGCAGAGCTT
	CCTCTTCCGTTGAGGGCCGCCAGCTGAAGCTTCG
C16-P1-left-50 bp	CAAGGGGTAGCGGCATCGCCTAGTAGCACTGTGGGGGGAAGAATACAACGGGCCGCCAG
	CTGAAGCTTCG
C16-P1-right-50 bp	AAACATAGAAGGTGCTGGCTGCATCTCGCAGAGCTTCCTCTTCCGTTGAGGGCCGCCAGC
	TGAAGCTTCG
C16-P1-left-25 bp	GCACTGTGGGGGGAAGAATACAACGGGCCGCCAGCTGAAGCTTCG
C16-P1-right-25 bp	TCGCAGAGCTTCCTCTTCCGTTGAGGGCCGCCAGCTGAAGCTTCG

For probe preparation

Probe 1-forward	GGATTTATTGGATTGGCACG
Probe 1-reverse	CTGCAGCGTACGAAGCTTCAGCTGGCGGCCACGGATGAGTCAAACCATAG
Probe 2-forward	AACGCAAATACGAGCGAAAG
Probe 2-reverse	CTCTGCCGCACTAAGATATT
Probe 3-forward	ATTGAAAGCACTCTAGCTCG
Probe 3-reverse	ATTTAGACGCCTCAGATGAC
Probe 4-forward	TATGCGTTGTTCTTCAGGCA
Probe 4-reverse	CCTCTCTTATCAATTGGGCT
Probe 5-forward	GGCTCGTTTGGATCAGTTTT
Probe 5-reverse	ACAACTGATGTAGCTGTTCC
Probe 6-forward	GCGGATCAAAAAACCATTCC
Probe 6-reverse	ACGGCAGCATTATTAACAGC
Probe 7-forward	GTGCTGCTAATAGGATTCGT
Probe 7-reverse	CATGTCGAACACTTTATGGC
Short CA primer	AGGCCACTAGTGGATCTGAT
